# Irreducible knee dislocation: improved clinical outcomes of open and arthroscopic surgical treatment. A systematic review of the literature

**DOI:** 10.1007/s00590-023-03781-x

**Published:** 2023-11-23

**Authors:** Fortunato Giustra, Francesco Bosco, Virginia Masoni, Marcello Capella, Giorgio Cacciola, Salvatore Risitano, Luigi Sabatini, Lawrence Camarda, Alessandro Massè

**Affiliations:** 1 Department of Orthopaedics and Traumatology, Ospedale San Giovanni Bosco di Torino - ASL Città di Torino, 10154 Turin, Italy; 2 Department of Orthopaedics and Traumatology (DiChirOnS), University of Palermo, Palermo, Italy; 3 Department of Orthopaedics and Traumatology, G.F. Ingrassia Hospital Unit, ASP 6, 90131 Palermo, Italy; 4 Department of Orthopaedics and Traumatology, University of Turin, CTO, 10125 Turin, Italy; 5 Ortopedia Protesica e Robotica–Humanitas Gradenigo, 10153 Turin, Italy

**Keywords:** Irreducible knee dislocations, IKDs, Reduction, Surgery, Arthroscopy, Systematic review

## Abstract

**Purpose:**

Irreducible knee dislocations (IKDs) are a rare rotatory category of knee dislocations (KDs) characterized by medial soft tissue entrapment that requires early surgical treatment. This systematic review underlines the need for prompt surgical reduction of IKDs, either open or arthroscopically. It describes the various surgical options for ligament management following knee reduction, and it investigates their respective functional outcome scores to assist orthopedic surgeons in adequately managing this rare but harmful KD.

**Methods:**

A comprehensive search in four databases, PubMed, Scopus, Embase, and MEDLINE, was performed, and following the PRISMA guidelines, a systematic review was conducted. Strict inclusion and exclusion criteria were applied. Studies with LoE 5 were excluded, and the risk of bias was analyzed according to the ROBINS-I tool system. This systematic review was registered on PROSPERO. Descriptive statistical analysis was performed for all data extracted.

**Results:**

Four studies were included in the qualitative analysis for a total of 49 patients enrolled. The dimple sign was present in most cases. The surgical reduction, either open or arthroscopically performed, appeared to be the only way to disengage the entrapped medial structures. After the reduction, torn ligaments were addressed in a single acute or a double-staged procedure with improved functional outcome scores and ROM.

**Conclusions:**

This systematic review underlines the importance of promptly reducing IKDs through a surgical procedure, either open or arthroscopically. Moreover, torn ligaments should be handled with either a single acute or a double-staged procedure, leading to improved outcomes.

**Level of evidence:**

IV.

## Introduction

Knee dislocations (KDs) are rare events with an incidence of up to 0.20% among all orthopedic trauma, and they are assumed to be underestimated because, in most cases, they undergo a spontaneous reduction [1–4].

Irreducible knee dislocations (IKDs), first described in the early twentieth century by Hull et al. [5] and later deepened by Ruppanner et al. [6], account for about 4% of all KDs representing a unique subcategory of KDs with specific features.

The traumatic mechanism is usually a posterolateral dislocation with a valgus force applied to a flexed knee [7–9]. The valgus force separates the medial femoral condyle from the tibial plateau, with the medial structures, especially the medial collateral ligament (MCL) along with the capsule and the retinaculum that remain entrapped in the joint as the energy dissipates and the knee partially tries to reduce back [7–9]. These structures are hardly extricated with closed maneuvers, so IKDs require early surgical reduction to avoid potential skin and soft tissue necrosis [7–10]. At inspection, the medial soft tissue entrapment presents as a “dimple sign” or “pucker sign” that, when present, is pathognomonic of IKDs [7–10]. Traditionally, surgical reduction with an open approach has been adopted [7–9]. However, Dubberley et al. [11] introduced the arthroscopic reduction procedure. Malik et al., in 2022, proposed the most recently updated algorithm for the management of IKDs [8]. They suggested that following the mandatory surgical reduction, an assessment of the cruciate and collateral ligaments should be done, with the reconstruction or repair of MCL along with the capsule and the retinaculum at the initial surgery [8]. The other structures are managed in a one- or a double-staged procedure [8]. Despite this, several authors described their strategies and ligament management experience. However, due to the heterogeneity and complexity of these lesions, controversies still exist regarding the best treatment, and strong conclusions cannot be set [9, 12–16].

This systematic review (SR) aims, in agreement with the most recent SR, to underline the need for prompt surgical reduction of IKDs, either open or arthroscopically performed. Moreover, it describes the various surgical options for ligament management following knee reduction. It also analyzes their respective clinical and functional outcomes to help orthopedic surgeons adequately struggle with this uncommon and insidious knee injury.

## Methods

### Research question

The Preferred Reporting Items for Systematic Reviews and Meta-Analyses (PRISMA) flow diagram was used to perform the research and select the studies included in this systematic review [17]. Two independent authors (VM and FB) searched and reviewed the final included articles to avoid possible bias. In case of discrepancy, a third author (FG) was consulted.

### Study selection and search strategy

A comprehensive literature search was conducted in four databases (PubMed, Scopus, Embase, and MEDLINE) with the following MeSH terms: [(irreducible knee dislocation) OR (posterolateral knee dislocation) OR (knee dislocation)].

**Fig. 1:**
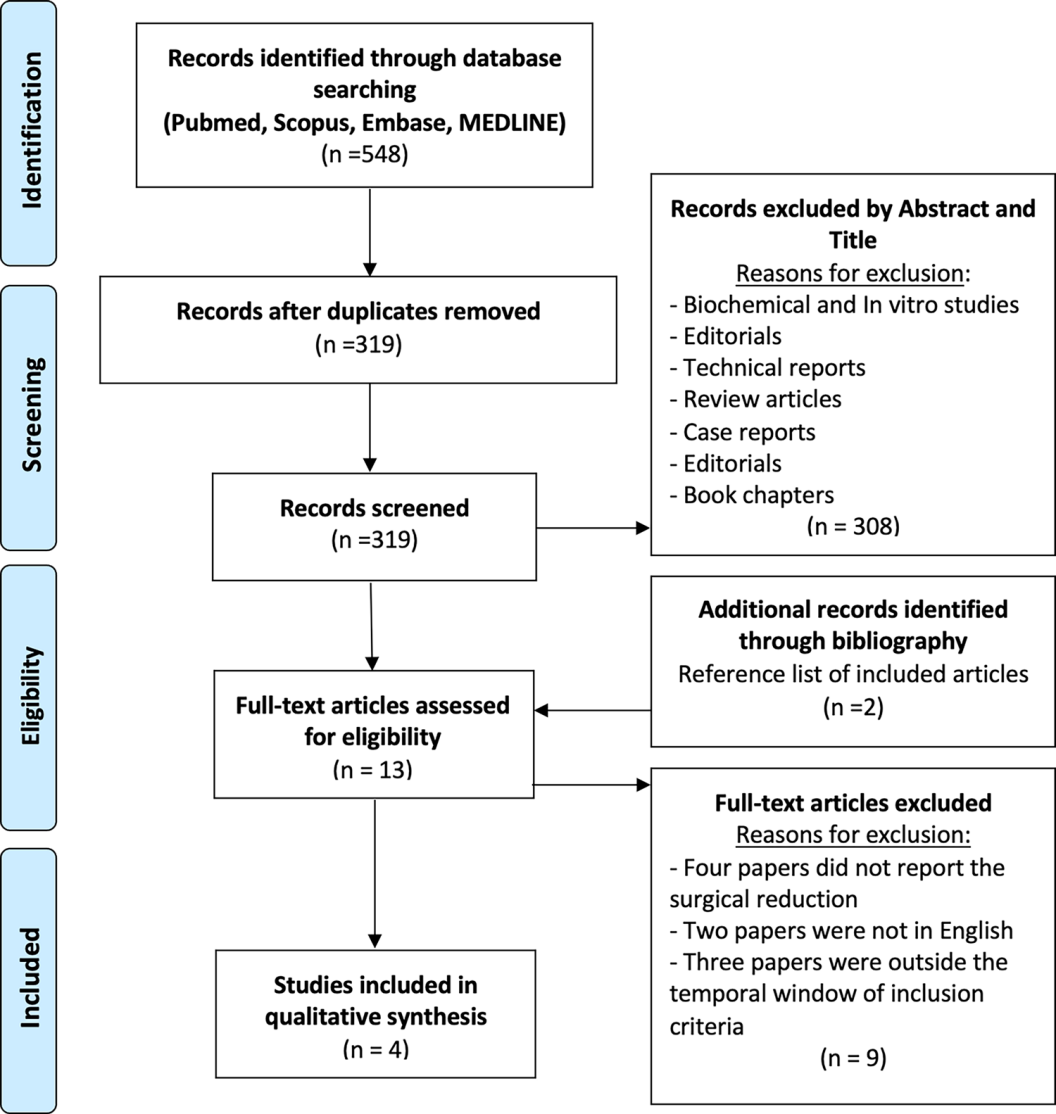
Preferred Reporting Items for Systematic Reviews and Meta-Analyses (PRISMA) flow diagram of articles included in the systematic review

The search ended on the 1st of September 2023. A total of 548 studies were identified in the extensive research. After eliminating duplicates, 319 articles were examined. Of these, 308 papers were excluded after title and abstract appraisal. Eleven papers underwent full-text evaluation with the addition of two articles after the bibliography of these studies was screened. A total of four clinical articles [12–15] dealing with irreducible knee dislocations surgically reduced, either open or arthroscopically, with subsequent ligament management were included in the systematic review for the final qualitative analysis. The PRISMA diagram is shown in Fig. 1.

### Inclusion and exclusion criteria

The included articles were written in English and published between January 2015 and September 2023 on human subjects only, whose patients had irreducible knee dislocation and underwent surgical reduction. This timeline was selected since surgical strategies, especially concerning ligament management, have been evolving rapidly, and the review aims to be the most updated as possible. Papers not reporting the description of the procedure adopted for the reduction were excluded. One of the articles selected included both acute and chronic IKD dislocation; all were considered in the analysis to increase the number of IKD cases. Biochemical and in vitro studies, technical reports, preclinical studies, review articles, case reports, editorials, and book chapters were excluded from the analysis.

### Data extraction

After a careful investigation, data were inserted in Excel spreadsheets by two authors autonomously and subsequently unified (VM and FB). The following study characteristics were extracted: authors and year of publication, type of study with levels of evidence (LoE), number of patients, sex, age, Body Mass Index (BMI), mechanism of injury, injured side, Schenck classification, follow-up time, lost to follow-up, time elapsed from injury to surgery, type of surgery for reduction (either open or arthroscopic), the surgical procedure for the management of the ligaments (one-stage, double-staged procedure and chronic/delayed), entrapped structures, type of medial collateral ligament (MCL) injury, specific complications, arterial and nerve injury, presence of the dimple sign, functional outcome scores assessed before and after surgery along with range of motion (ROM), postoperative KT-1000 arthrometer values, and clinical examination tests.

### Functional outcome scores and quantitative assessment of knee stability

Three patient-reported outcome measures (PROMs) were analyzed to assess functional outcomes [18]. The Lysholm score consists of 100 points, where 0 represents complete disability, and 100 represents best functional status. It includes eight items: limp, need of support when walking, locking, instability, pain, swelling, stair-climbing capacity, and squatting [19]. The International Knee Documentation Committee (IKDC) is a 100-point scoring system in which a higher score means better functional outcomes and less disability. It has three main elements: symptoms, sports activities, and knee function [20]. The Tegner score assesses the ability to practice sports activities and work. It is a one-item score with a scale from 0 to 10, where 0 stands for “disability pension” due to knee problems and 10 means ability to play competitive sports [21].

The KT-1000 arthrometer and Telos stress radiographs were used to assess knee stability quantitatively. The KT-1000 arthrometer is a device placed on the knee that measures the translation of the tibia relative to the femur to assess anteroposterior stability at different knee flexion angles [22]. The Telos stress device is an instrument placed on the lower limb that, under X-ray and stabilized pressure, measures anteroposterior translation and medial and lateral opening at different knee flexion angles [23]. For both the KT-1000 arthrometer and the Telos stress device, laxity was indicated as a side-to-side difference (SSD) compared to the healthy side according to the IKDC standard: normal 0–2 mm, nearly normal 3–5 mm, abnormal 6–10, severely abnormal > 10 mm [13, 15].

### Methodological quality assessment

**Fig. 2:**
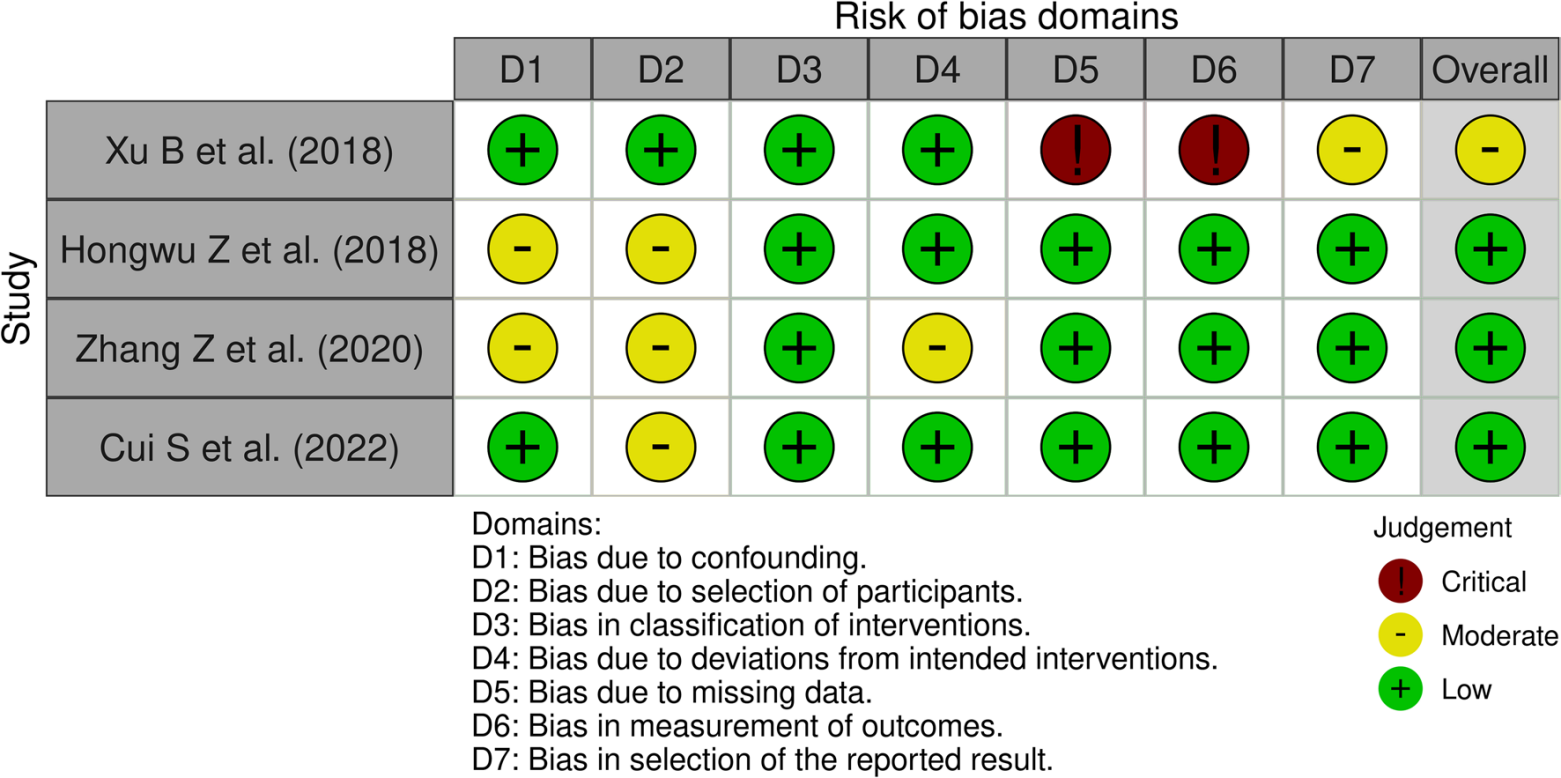
Risk of Bias In Non-randomized Studies—of Interventions (ROBINS-I) comprehensive table according to the Cochrane network. Evaluation of the risk of bias of the individual studies included in the systematic review according to the ROBINS-I assessment tool

Articles were graded according to the 2011 Oxford Centre for Evidence-Based Medicine LoE from 1 to 5, where LoE 1 represents the best quality design with the lowest risk of bias [24]. Retrospective studies with a level of evidence from 1 to 4 were included in this systematic review, while studies with LoE 5 were excluded. The included studies were analyzed with the Risk of Bias In Non-randomized Studies of Interventions (ROBINS-I) [25, 26] (Fig. 2). Two authors (VM and FB) used this tool, while a third author (FG) contributed to resolving any uncertainties. All authors contributed substantially to the conception and design of the study, manuscript drafting, final editing, and data acquisition. All authors approved the final version of the article. This systematic review was registered in the International Registry of Systematic Reviews (PROSPERO), CRD42022343488 [27].

### Statistical analysis

Statistical analysis was performed with R software, version 4.0.5 (2020; R Core Team, Vienna, Austria). Descriptive statistical analysis was performed for all data extracted from the included studies. For continuous variables, mean values were calculated or extracted with a measure of variability as standard deviation (SD) or range (minimum–maximum). For categorical variables, the absolute number and frequency distribution were calculated.

## Results

A total of 49 patients (32 (65.3%) males and 17 (34.7%) females) with IKDs were included in the systematic review. The mean age at the time of injury was 44.5 years old. Approximately half of the IKDs were caused by high energy and the other half by low-energy mechanisms. The study type, demographic characteristics, injury details, and follow-up time of the included articles are shown in Table 1. Surgical management regarding the reduction procedure and ligament strategies is reported in Table 2. Complications associated with IKDs are listed in Table 3. PROMs and ROM values before and after surgery are shown in Table 4. Finally, when reported, postoperative KT-1000 arthrometer assessment and physical examination values are presented in Table 5.

**Table 1:** Demographic data of patients included in the systematic review with associated study details

Authors and publication year	Study type	LoE	Sample Size, N°	Sex, N°		Age, y.o	BMI	Mechanism of injury, N°	
				M	F	Mean ± SD (range)	Mean ± SD (range)	Low	High
Xu et al. 2018 [12]	RS	IV	6	5	1	51.2 ± 9.7 (38–67)	/	0	6
Hongwu et al. 2018 [13]	RS	IV	13	8	5	37.8 ± 8.0 (27–56)	/	4	9
Zhang et al. 2020 [14]	RS	III	13	8	5	46.7 (21–68)	23.6 ± 2.1	7	6
Cui et al. 2022 [15]	PS	III	17	11	6	42.5 ± 8.9 (29–60)	25.9 ± 2.1 (22–29.1)	14	3

Authors and publication year	Injured Side, N°		Schenck classification, Type						Follow-up time, months	Lost to follow-up, N°
	L	R	KDI	KDII	KDIIIM	KDIIIL	KDIV	KDV	Mean ± SD (Range)	
Xu et al. 2018 [12]	3	3	0	0	3	0	2	1	/	/
Hongwu et al. 2018 [13]	7	6	/	/	/	/	/	/	32.6 ± 7.1 (24–46)	2
Zhang et al. 2020 [14]	/	/	0	0	13	0	0	0	42.9 (28–78)	1
Cui et al. 2022 [15]	/	/	0	0	17	0	0	0	11.2 ± 2.5 (6–14)	3*

*RS*: Retrospective; *PS*: Prospective; *LoE*: Level of Evidence; N°: Number of cases; *M*: male; *F*: female; y.o.: Years old; *BMI*: Body mass index; *SD*: Standard deviation; *L*: left; *R*: right; *KD*: Knee Dislocation; /: not reported/not mentioned in the paper.

*Three patients were lost at 1-year follow-up, while all patients were present at 0.5-year follow-up. All the values are approximated at one decimal

**Table 2:** Surgical management of IKD

Authors and publication year	Time from injury to surgery, days	Surgical reduction procedure type			Ligament managements		
	Mean ± SD (range)	Open, N°	Arthroscopy, N°	First arthroscopy attempt, then open, N°	One-stage acute procedure	Double-staged procedure	Delayed (Chronically management > 3 weeks)
Xu et al. 2018 [12]	≤ 1*	6	0	0	6	0	0
Hongwu et al. 2018 [13]	1.8 ± 0.8 (1–3)	0	13	0	13	0	0
Zhang et al. 2020 [14]	**	0	0	13	6	0	7
Cui et al. 2022 [15]	0.8 ± 0.9 (0–3)	17	0	0	0	17	0

N°: Number of cases; /: not reported/not mentioned in the paper

*Surgery performed on the admission day

**Acute treatment within ( ≤) 3 weeks in six cases and chronic treatment after ( >) 3 weeks in seven cases

**Table 3:** Trapped structures and complications associated with IKD

Authors and publication year	Trapped structures, N°					Type of MCL injury, N°			
	MR	C	MCL	VM	MM	F	T	M	PO
Xu et al. 2018 [12]	3	/	2	2	0	/	/	/	/
Hongwu et al. 2018 [13]	13	13	/	/	/	/	/	/	/
Zhang et al. 2020 [14]	/	/	/	/	/	9	0	4	0
Cui et al. 2022 [15]	/	/	/	/	/	6	4	6	1

Authors and publication year	Complications, N°							Neurovascular assessment, N°		Dimple sign, N°
	Meniscus tear	CS	Patellar dislocation	Soft tissue necrosis	Knee stiffness	KH	KFC	Neural lesion	Vascular	
Xu et al. 2018 [12]	/	/	/	/	/	/	/	1	0	6
Hongwu et al. 2018 [13]	/	0	/	0	1	/	/	0	0	/
Zhang et al. 2020 [14]	/	/	/	2*	/	/	/	1	0	6
Cui et al. 2022 [15]	/	/	/	/	0	3	3	0	0	**

*MR*: Medial retinaculum; *C*: Capsule; *MCL*: Medial collateral ligament; *VM*: Vastus medialis; *MM*: Medial meniscus; *F*: Femoral attachment; *T*: Tibial attachment; *M*: Mid-substance lesion; *PO*: Peel-off; *CS*: Compartment syndrome, *KH*: Knee hemarthrosis; *KFC*: Knee flexion contracture; N°: Number of cases; /: not reported/not mentioned in the paper

*Only in the chronic IKD group

**Referred in the text as “sometimes”

**Table 4:** Patient reported outcome measures (PROMs) and range of motion (ROM) before and after IKD treatment

Authors and publication year	PROMs						ROM, Mean ± SD (range)	
	Lysholm score, Mean ± SD (range)		Tegner score, Mean ± SD (range)		IKDC score, Mean ± SD (range)			
	Pre-op	Post-op	Pre-op	Post-op	Pre-op	Post-op	Pre-op	Post-op
Xu et al. 2018 [12]	/	/	/	/	/	/	/	/
Hongwu et al. 2018 [13]	1.5 ± 2.4	83.5 ± 6.6	0.0 ± 0.0	4.8 ± 0.9	8.6 ± 1.8	75.4 ± 3.9	/	2.7 ± 5.6 to 132.7 ± 11.7
Zhang et al. 2020 [14]	/	79.2 (60–95)	/	4.5 (4–6)	/	78.6 (60.9–95.4)	/	118.1 (90–140)
Cui et al. 2022 [15]	/	84.5 ± 4.5	/	/	/	79.0 ± 5.7	/	−3.1 ± 2.5 to 138.1 ± 8.9

*PROMs*: Patient-reported outcome measures; *SD*: standard deviation; *ROM*: range of motion; *IKDC* International Knee Documentation Committee. All the values are approximated at one decimal

**Table 5:** KT 1000 arthrometer assessment and physical examination at final follow-up.

Authors and publication year	KT 1000 arthrometer assessment at final follow-up									
	(30° forward shift or 25° isolated anteroposterior translation)					(70° backward shift or 70° total anteroposterior translation)				
	SSD mm, Mean ± SD	0–2 mm, N°	3–5 mm, N°	6–10 mm, N°	> 10 mm, N°	SSD mm, Mean ± SD	0–2 mm, N°	3–5 mm, N°	6–10 mm, N°	> 10 mm, N°
Xu et al. 2018 [12]	/	/	/	/	/	/	/	/	/	/
Hongwu et al. 2018 [13]	1.7 ± 0.9	12	1	0	0	2.2 ± 1.6	10	3	0	0
Zhang et al. 2020 [14]	/	/	/	/	/	/	/	/	/	/
Cui et al. 2022 [15]	/	12	0	0	2	/	11	3	0	0

Authors and publication year	Physical examination at final follow-up													
	Lachman test, N°		Pivot shift test, N°		Posterior drawer test, N°		Varus stress test, N°				Valgus stress test, N°			
	–	± or +	–	± or +	–	± or +	–		± or +		–		± or +	
							NS	30°	NS	30°	NS	30°	NS	30°
Xu et al. 2018 [12]	/	/	/	/	/	/	/	/	/	/	/	/	/	/
Hongwu et al. 2018 [13]	12	1	12	1	11	2	/	13	/	0	/	10	/	3
Zhang et al. 2020 [14]	/	/	/	/	/	/	/	/	/		/	/	/	/
Cui et al. 2022 [15]	14	0	14	0	14	0	13	/	1	/	14	/	0	/

*SD*: Standard deviation; *SSD*: Side-to-side difference; *NS*: Not specified; N°: Number of cases; *mm*: Millimeters. −: negative test, + : Positive test; ± : Uncertain test. All the values are approximated at one decimal

## Discussion

The most important finding of this SR is that IKDs require a prompt surgical reduction, either open or arthroscopically performed, since closed maneuvers cannot disengage the medial entrapped structures, and they potentially lead to an increased risk of soft tissues and neurovascular damage. This observation perfectly aligns with the most recent algorithm proposed by Malik et al. [8]. Furthermore, this SR provides the attending orthopedics with the various surgical options concerning ligament repair/reconstruction following knee reduction, and it reports improved functional outcome scores after them. Indeed, even if a meta-analysis could not be conducted, all the PROMs as the Tegner score, the Lysholm score, and the IKDC improved after the various surgical strategies adopted along with the knee ROM.

IKDs represent a unique, rare rotatory subcategory of KDs [1–4, 7, 8, 10, 28]. As described by Malik et al. [8], most of them occurred through a posterolateral displacement, and according to the Schenck classification, they start from KD IIIM as a grade of severity [12, 14, 28]. With KD type III reported as the most common type in the entire KD literature, this systematic review agrees since KD III was the major injury pattern described [4, 12, 14, 28].

### Demographics and mechanism of injury

Two-thirds of the patients were males, and the mean age of the population investigated corresponds to the mean age reported by Malik et al. [8]. In the literature, two main mechanisms of injury are described at the base of KDs as either low or high-energy trauma [1–4, 10]. It is well known that KDs overall are most commonly due to high-energy trauma, such as road traffic accidents [1–4], and this is consistent with the IKDs group by Xu et al., whose patients were admitted after high-energy trauma, especially motor accidents [12]. Nonetheless, in this SR, half of the patients sustained a low-impact trauma as a fall, which aligns with the most recent SR of Malik et al., supporting low energy mechanisms as a cause of IKDs [8].

### Clinical presentation, initial assessment, and complications

As with the entire KD entity, when IKDs are suspected, careful and detailed neurovascular assessment should be performed, and compartment syndrome should be ruled out [1–4, 10].

**Fig. 3:**
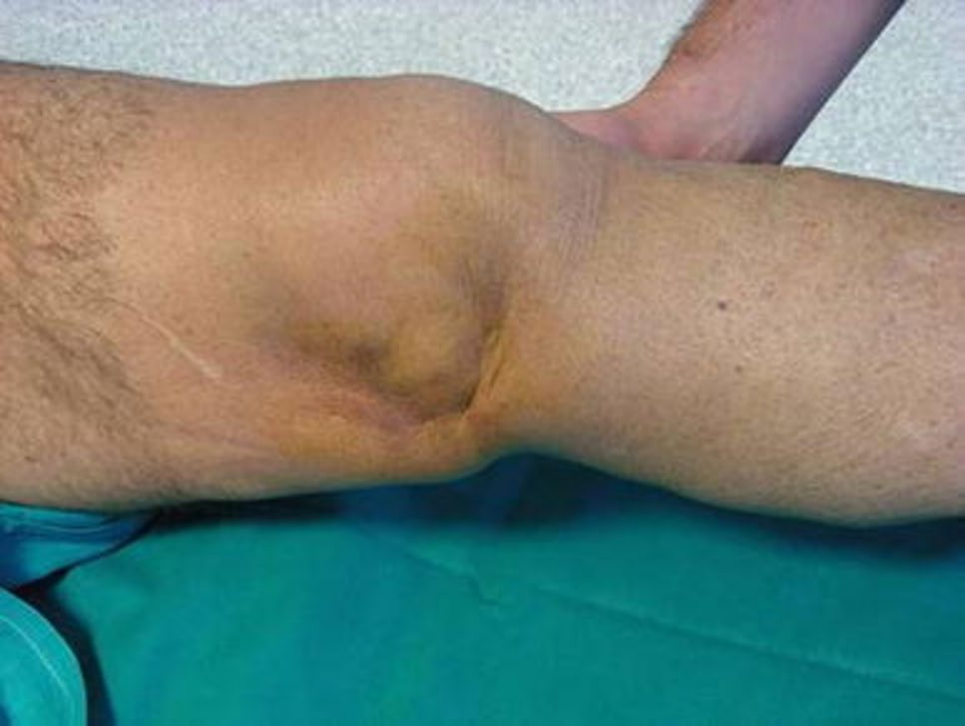
Dimple sign: skin invagination and entrapment in the medial joint space reported by Bistolfi et al. [16]

In aiding the diagnosis of IKDs, a pathognomonic medial “pucker” or “dimple sign” should be looked for [8, 10–12] (Fig. 3). This phenomenon arises from the entrapment of the medial structures during the dislocation [7, 8, 10–12]. The medial femoral condyle usually buttonholes the medial structures, and it becomes subcutaneously visible with the skin below invaginated inward [7–12]. However, this sign is not necessary for the diagnosis described by Durakbaşa et al., where entrapment of the medial meniscus did not manifest as a dimple [29]. Malik et al. described this sign in 70% of patients [8]. In this SR, percentages were not calculated since not all the Authors reported it [13]; nonetheless, Xu et al. [12] outlined it in all the patients, and Zhang et al. [14] reported it in all the acute IKD cases.

A unique complication of IKDs, related to medial tissue invagination, is skin and soft tissue necrosis [7–10, 13, 28]. This complication could be potentially avoided when urgent reduction is performed [7–10, 13, 28]. Even if this aspect was not cited in some of the papers reviewed, Zhang et al. underlined some cases of tissue necrosis in the chronic subgroup [14].

While it is well known that KDs pose at risk the common peroneal nerve (around 20%) and the popliteal artery (around 19%) [1–4, 10], there are limited data regarding these complications in IKDs. This systematic review shows no vascular injuries or compartment syndrome cases were present. One case of both peroneal and tibial nerve palsies [12] and one case of partial sensory loss of the common peroneal were reported [14]. These findings are consistent with the most recent SR reporting the rate of neurovascular injuries for the IKDs category is lower than for KDs [8]. Malik et al. suggested that the low rate of neurovascular injuries for IKDs is derived from the low kinetic causative mechanism since the valgus force is less likely to strain the vessels and nerves that lie anatomically more laterally in the popliteal fossa [8]. Mentioning other complications, some authors [12] did not report them, but when present, knee stiffness and knee flexion contracture were the most common [15].

### Entrapped structures and pattern of MCL injuries

**Fig. 4:**
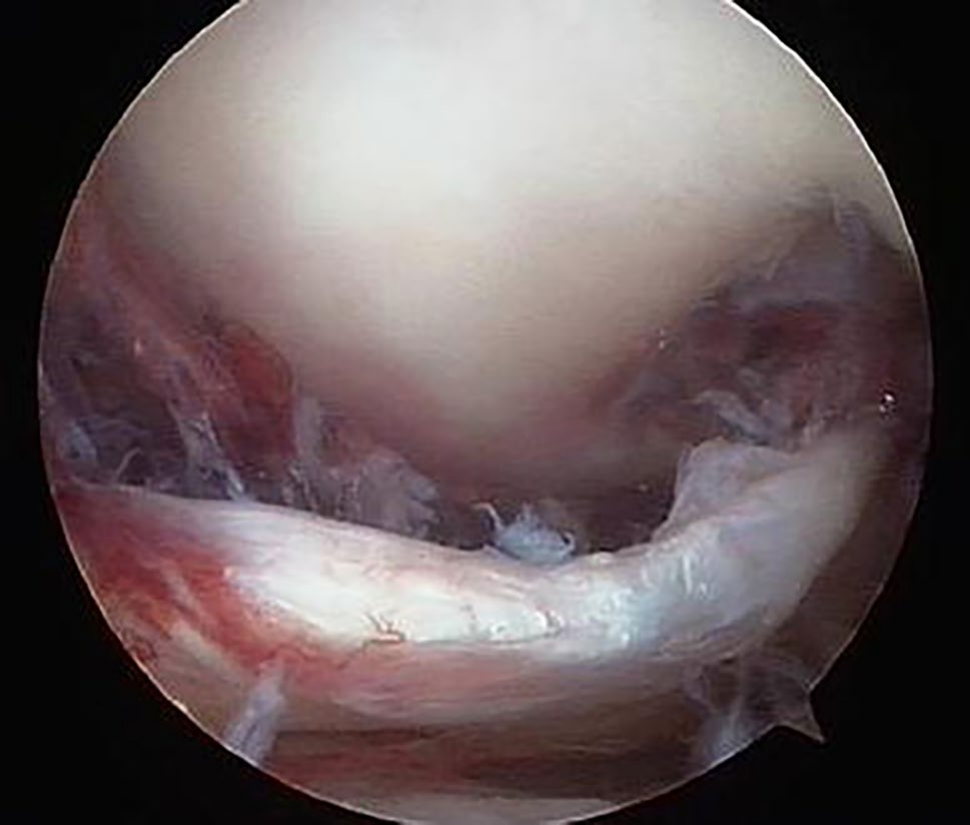
Knee arthroscopy showing intra-articular dislocation of the vastus medialis reported by Bistolfi et al. [16]

Of particular interest in the literature are the medial structures entrapped [8]. The most frequently torn structures are the medial capsule with the retinaculum and MCL, but other elements, such as the vastus medialis or medial meniscus, could be involved [6–9, 16, 29] (Fig. 4). Different patterns of MCL injuries exist, but in this SR, the majority are at the femoral attachment and the mid-substance. This evidence is reported by Cui et al. [15] and by Zhang et al. [14], and it follows the biomechanical study of Wijdicks et al., underlining that stronger loads can be sustained by superficial and deep MCL tibial attachment [30].

### Surgical strategies

Nowadays, following the algorithm proposed by Malik et al. [8], prompt surgical reduction seems to be the only solution to reduce IKDs by mechanically disengaging the entrapped medial structures. Pache et al., in a recent case report, agreed not to waste time attempting a closed reduction more than once since, in most cases, this fails [9]. Moreover, trials of closed reduction maneuvers risk an increase in soft tissue necrosis and neurovascular damage [7–10].

### Open versus arthroscopic reduction procedure

In the literature, both an open procedure and an arthroscopic approach have been described to disengage the entrapped medial structures [7–9, 11–15]. Traditionally, an open approach to the knee was used [8, 12], and the first who introduced the arthroscopic procedure to disengage the medial structures was Dubberley et al. [11] in two patients. Hongwu et al., in their case series, excluded patients undergoing open reduction and described the surgical pearls and pitfalls for a successful arthroscopic reduction [13].

What emerged from the most recent SR by Malik et al. is that the traditional open approach, especially a medial arthrotomy, is the most frequently used, with arthroscopic only reduction performed in less than 20% of cases [8]. However, as already performed by some Authors [14], a diagnostic arthroscopy and attempted arthroscopic reduction followed by an eventual open approach in case of failure is suggested by Malik et al. [8]. Nonetheless, recent authors such as Cui et al. [15] in 2022 and Pache et al. [9] in 2023 still used an open approach.

### Single acute versus double-staged procedure

Once the knee has been reduced, surgeons should address the torn soft tissues [1–4, 7–10, 31]. Three surgical strategies have been reported in the literature concerning multi-ligaments knee injuries (MLKI): an acute repair/reconstruction in one stage procedure, a double-staged procedure with acute repair/reconstruction of the extra-articular structures, followed by a rehabilitation period and pivot reconstruction once full range of knee motion is regained, and a delayed reconstruction with surgery more than three weeks after the injury [1–4, 7–10, 14, 31]. As reported by Howell et al. [1] and Ng et al. [3] in MLKI, nowadays, there is a consensus towards an early surgical intervention since the torn structures are still definable without significant scarring and retraction so a direct repair could be performed [3]. In accordance, Zhang et al. reported better Lysholm and IKDC scores for the acutely treated group [14]. Moreover, the acute treatment gives a better restoration of knee kinematics and reduces further chondral and meniscal damage [3]. The drawbacks well-reported are the risk of joint stiffness and arthrofibrosis, which could be impeded with an intense rehabilitation program [3, 31].

Ng et al. [3] in overall KDs and Cui et al. [15] in IKDs support a staged reconstruction since they agree that an intermediate aggressive rehabilitation facilitates the recovery of ROM and knee function.

On the contrary, Xu et al. [12] and Hongwu et al. [13] managed the IKDs in one stage. Concerning functional outcomes, they were acceptable in either the single acute or the staged procedure, respectively [12–15]. Regarding the pivot reconstruction, Cui et al. showed increased anteroposterior stability after stage two surgery [15]. On the contrary, Bistolfi et al. [16], in their case report, decided not to perform pivot reconstruction, given the minimal clinical residual instability, and they argued that cruciate ligament reconstruction could be avoided in the elderly or those not engaged in high professional sports activity [16]. This evidence is in line with the most recent SR since these complex lesions should be strictly followed in time, and the treatment should be tailored to each patient, with not all requiring ligament reconstructions [8].

### PROMs and ROM after surgical management

Concerning a previous SR where only the ROM and IKDC scores were analyzed [8], in this SR, the Lysholm score and the Tegner score were considered. All PROMs, when reported, along with ROM, improved after surgery independently of the strategy adopted [12–15].

Hongwu et al. reported a significant improvement in the IKDC score, the Lysholm score, and the Tegner score postoperatively (p-value = 0.001) [13]. Cui et al. showed an increase in the Lysholm score and IKDC after the second surgical procedure [15]. Zhang et al. expressed good functional outcomes at the follow-up time [14].

Regarding ROM, flexion of more than 100° was reported by three of the studies included [13–15], with Cui et al. [15] and Hongwu et al. [13] describing a flexion of approximately around 130°.

### Strengths and limitations

This SR has strengths and limitations. The present SR enhances the algorithm proposed by Malik et al. [8] since all IKDs, when diagnosed, underwent prompt surgical reduction. Moreover, it implements the previous SR by reporting improved functional outcomes following ligament management.

However, some limitations persist and should be evaluated. First, a few intermediate-quality studies are included in the analysis, mainly related to IKDs rarity. Most of them are retrospective case series; only one study included was a prospective case series. All presented a small sample size and a short follow-up period. Second, several surgical techniques were adopted regarding ligament management following the reduction with different rehabilitation programs used, which could create bias in evaluating the outcomes. In addition, some authors did not report specific details, such as the chondral or meniscal lesions analyzed by Malik et al. [8]. Third, it was not possible to perform a meta-analysis of the results presented in the individual studies because of their high heterogeneity.

Finally, a meta-regression analysis, the application of the GRADE approach, and specific sensitivity analysis were not accomplished due to incomplete reporting variables and the low quantity of data of the studies included.

Concerning implications for future research, higher-quality studies, with control groups and RCTs, will be necessary to sort out the best surgical technique for the reduction and subsequent ligament management.

## Conclusion

IKDs are rare events where skin and soft tissue remain entrapped medially following a rotatory mechanism. Since they could present subtly, their pathognomonic “dimple sign” should be examined. Early surgical treatment is mandatory to reduce dislocation, and trials of closed maneuvers should be avoided since they increase the risk of medial tissue necrosis. Both the traditional open approach and the more recent arthroscopic procedure could be used to reduce this condition. Following reduction, torn ligament should be managed, either in a single or a staged procedure, with good clinical and functional outcomes achieved despite the complexity of these lesions. Higher-quality studies, such as RCT or multicenter studies, will be necessary to compare and define the best surgical approach.

## Data Availability

The dataset analyzed in this study is available from the corresponding author on reasonable request.

## Abbreviations

KD: Knee dislocation
IKD: Irreducible knee dislocation
MCL: Medial collateral ligament
SR: Systematic Review
PRISMA: Preferred reporting items for systematic reviews and meta-analyses
LoE: Level of evidence
PROM: Patient reported outcome measures
IKDC: International knee documentation committee
TAS: Tegner activity score
ROBINS-I: Risk of bias in non-randomized studies of interventions
PROSPERO: International prospective register of systematic reviews
SD: Standard deviation
SSD: Side-to-side difference
ROM: Range of motion
MM: Millimeters
CT: Computed tomography
MRI: Magnetic resonance imaging
MLKI: Multi-ligament knee injuries
BMI: Body mass index
RCTs: Randomized control trials
